# Cost analysis of Periodontitis management in public sector specialist dental clinics

**DOI:** 10.1186/1472-6831-14-56

**Published:** 2014-05-20

**Authors:** Tuti Mohd-Dom, Rasidah Ayob, Amrizal Mohd-Nur, Mohd R Abdul-Manaf, Noorlin Ishak, Khairiyah Abdul-Muttalib, Syed M Aljunid, Yuhaniz Ahmad-Yaziz, Hanizah Abdul-Aziz, Noordin Kasan, Ahmad S Mohd-Asari

**Affiliations:** 1 Department of Dental Public Health, Faculty of Dentistry, Universiti Kebangsaan Malaysia, Kuala Lumpur, Malaysia; 2 Oral Health Division, Ministry of Health, Malaysia, Putra Jaya, Malaysia; 3 International Centre for Casemix and Clinical Coding, Universiti Kebangsaan Malaysia Medical Centre, Kuala Lumpur, Malaysia; 4 Department of Community Health, Faculty of Medicine, Universiti Kebangsaan Malaysia, Kuala Lumpur, Malaysia

## Abstract

**Background:**

The objective of this paper is to quantify the cost of periodontitis management at public sector specialist periodontal clinic settings and analyse the distribution of cost components.

**Methods:**

Five specialist periodontal clinics in the Ministry of Health represented the public sector in providing clinical and cost data for this study. Newly-diagnosed periodontitis patients (N = 165) were recruited and followed up for one year of specialist periodontal care. Direct and indirect costs from the societal viewpoint were included in the cost analysis. They were measured in 2012 Ringgit Malaysia (MYR) and estimated from the societal perspective using activity-based and step-down costing methods, and substantiated by clinical pathways. Cost of dental equipment, consumables and labour (average treatment time) for each procedure was measured using activity-based costing method. Meanwhile, unit cost calculations for clinic administration, utilities and maintenance used step-down approach. Patient expenditures and absence from work were recorded via diary entries. The conversion from MYR to Euro was based on the 2012 rate (1€ = MYR4).

**Results:**

A total of 2900 procedures were provided, with an average cost of MYR 2820 (€705) per patient for the study year, and MYR 376 (€94) per outpatient visit. Out of this, 90% was contributed by provider cost and 10% by patient cost; 94% for direct cost and 4% for lost productivity. Treatment of aggressive periodontitis was significantly higher than for chronic periodontitis (t-test, P = 0.003). Higher costs were expended as disease severity increased (ANOVA, P = 0.022) and for patients requiring surgeries (ANOVA, P < 0.001). Providers generally spent most on consumables while patients spent most on transportation.

**Conclusions:**

Cost of providing dental treatment for periodontitis patients at public sector specialist settings were substantial and comparable with some non-communicable diseases. These findings provide basis for identifying potential cost-reducing strategies, estimating economic burden of periodontitis management and performing economic evaluation of the specialist periodontal programme.

## Background

Chronic diseases have arguably been shown to exert considerable economic impact on both health care systems and the individual patient [1,2]. Treatment of oral diseases, for instance, account for the fourth most expensive disease in many industrialised countries, and costs are often borne by patients’ out-of-pocket payments [3]. Periodontitis is an established and widespread chronic disease, yet its burden on healthcare costs remain largely neglected. While cost-related studies in oral healthcare are fewer compared to medical care, cost-of-illness (COI) studies of oral diseases are extremely rare [4-6]. Moreover, the majority of cost analysis studies in dentistry had focused on cost of running a whole public dental programme [7] or unit cost of providing a particular restorative [8-10], preventive [11] or diagnostic [12] procedures.

Most studies estimating costs of periodontal care focus on cost of specific periodontal treatment modalities [13-15] but not the cost of managing the whole spectrum of the disease itself. The COI study approach is a descriptive study that can provide information to measure economic burden of a disease using a prevalence-based or an incidence-based approach. Prevalence-based COI studies measure current economic burden of a disease in a given period, whereas incidence-based approach measures economic burden from the onset of disease until cure or death and involves estimating the lifetime costs of new cases which have their onset in a given period of time [16].

Studies employing cost-of-illness approach in periodontology are scarce; one such study is a Norwegian study which estimated lifelong cost of managing periodontitis in a specialist practice [4]. Another is on the cost-effectiveness of supportive periodontal care between specialist and generalist periodontal practice [17]. These studies however, had estimated costs based on recommended charges, third-party reimbursements or national expenditures. Hence they are likely to provide an underestimation of costs, and in the absence of real cost data it is not possible to analyse distribution of cost components and identify cost-saving strategies.

In Malaysia, economic studies related to dental care have yet be extended in the area of periodontology [10] in spite of the decline in periodontal health of Malaysian adults [18]. In the light of rising healthcare costs, a cost-of-illness (COI) study in periodontitis will provide the needed cost estimates that result from the condition. These estimates among others may be used to justify intervention programmes, assist in allocation of resources and provide an economic framework for programme evaluation [19]. The purpose of this study was to quantify the cost of periodontitis management at public sector specialist periodontal clinic settings using a COI approach and to analyse the distribution of the cost components.

## Methods

### Ethics

Permission to conduct the study was obtained from the Institutional Review Boards of the Ministry of Health, Malaysia and Universiti Kebangsaan Malaysia.

### Clinic selection

The sampling frame for clinic selection comprised all eighteen Ministry of Health specialist periodontal clinics located throughout the country. Selection of five participating clinics was made based two stages: first stage is to identify five geographical zones in Peninsular Malaysia (north, west, east, central and south); second stage was based on random sampling of clinics in each zone which met the following selection criteria: operate at health centres or polyclinics and possess adequate clinical and administrative records. Specialist dental clinics in East Malaysia were excluded due to logistic reasons such as the larger geographical area served by the public sector and were not as accessible by the public as the clinics in Peninsular Malaysia due to the lower dentist to population ratio. Generally there is a difference between oral health status and health-seeking behaviour between the population of Peninsular Malaysia and East Malaysia. Educational levels, income, lifestyle and culture are generally the same.

### Patient recruitment and one-year periodontal therapy

**Figure 1:**
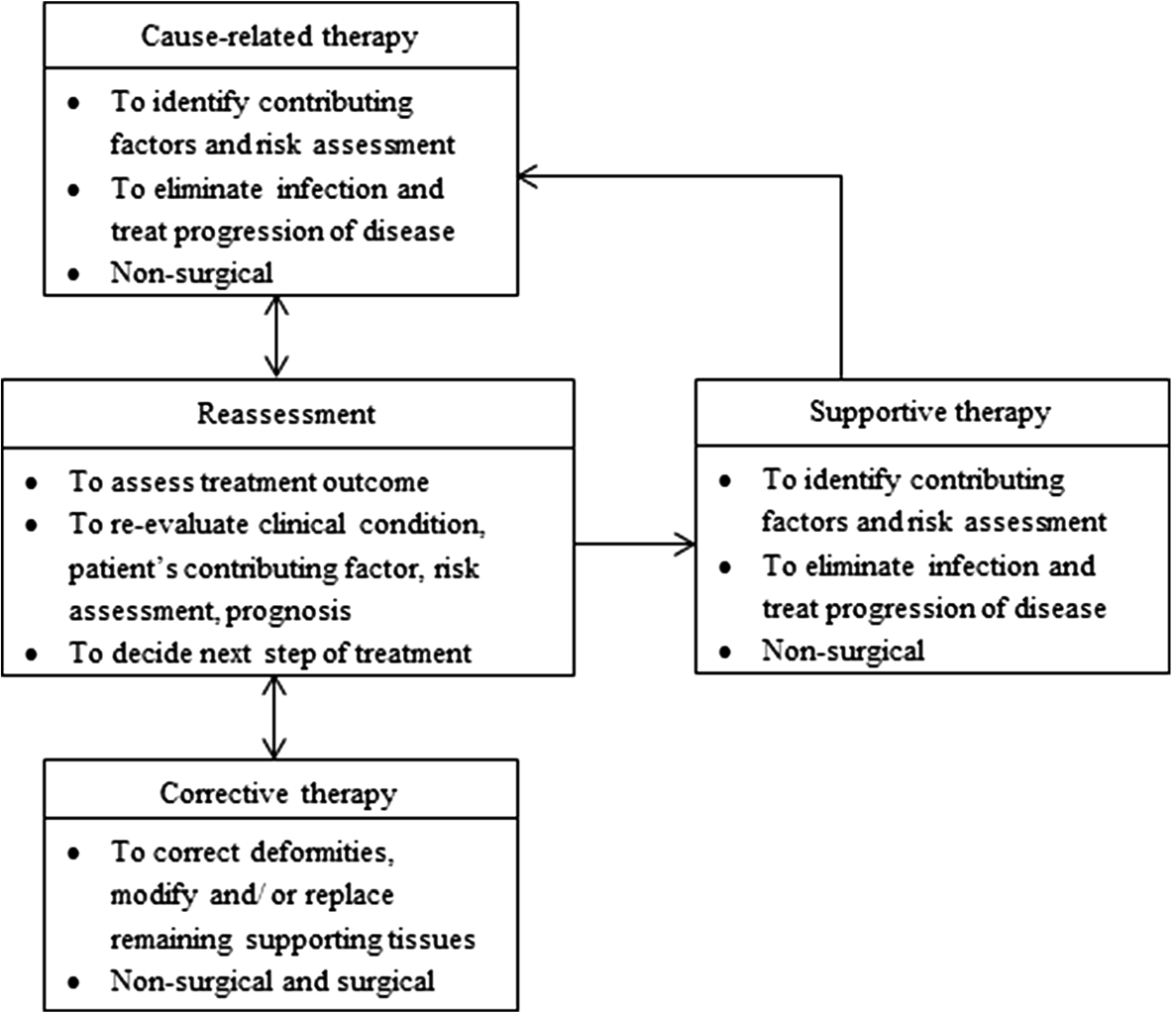
Phases of periodontal therapy.

Recruitment of newly diagnosed periodontitis patients seen at the five participating clinics started concurrently in November 2010. They were selected on a consecutive basis. The referral of these patients to the specialist clinics was based on the criteria that at least one sextant of the dentition presented with 4 mm periodontal pocket depth or more (code 3 or 4 of the Basic Periodontal Examination Index) during clinical examination. Patients must not have had any periodontal treatment (except gross scaling) within six months prior to commencement of study. Recruitment of these new patients ended in August 2011 with a total of 165 patients. Upon recruitment, they were provided the necessary dental treatment according to the phases of periodontal therapy within a period of twelve months (Figure 1). All clinics were under the purview of the Ministry of Health, provided with similar equipment, dental materials and operating budget. It is assumed that interventions were based on the existing clinical practice guidelines although personal preferences of specialists would have affected the study. Decisions to perform surgery was based on specialists’ best clinical judgment but guided by the best evidence as in the clinical practice guidelines. Follow-up assessment for periodontal treatment received ended in August 2012.

### Cost analysis

**Figure 2:**
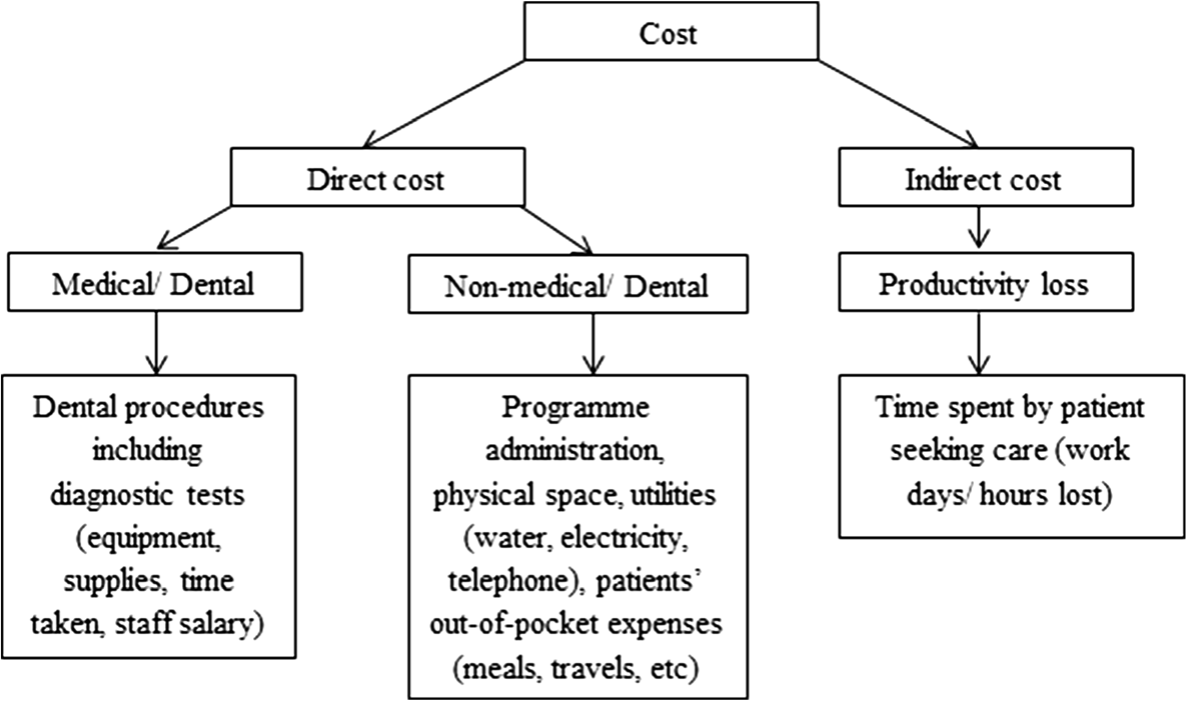
Components of cost analysis.

We performed the cost analysis according to methods proposed by Creese and Parker [20], Shepard et al. [21] and Drummond et al. [22]. We conducted the analysis from the societal perspective which includes the economic viewpoint of the provider, Ministry of Health, Malaysia, and the patients. All costs in the analysis are presented in Malaysian Ringgit (MYR) 2012. Components of cost analysis include both direct and indirect costs and are illustrated in Figure 2. Direct medical or specifically dental costs refer to resources consumed for dental and periodontal treatment performed for periodontitis patients in this study. The scope of costs included resources consumed for 30 procedures classified into diagnostics, non-surgical periodontal therapy and surgical interventions performed for periodontitis patients – these were determined by an expert panel discussion via a clinical pathway. Direct non-medical/ dental costs were those expended for programme administration, physical space, utilities (water, electricity, telephone) and patients’ out-of-pocket expenses for meals and travels. Indirect costs refer to productivity loss on the part of the patients due to time spent in seeking dental care. This was measured using the human capital approach.

Sources of data came from clinic, annual returns, administrative and financial record for year 2011, as well as observation of 60 patients undergoing various treatments. Cost of rehabilitative dental procedures such as restorations, endodontics, dentures, crown and bridgework was estimated using the 2012 Ministry of Health government subsidy rates [23] and the Malaysian Dental Association recommended scale of fees for year 2010 [24]. Patient diaries provided primary data for patient out-of-pocket expenditures and time taken off work, whenever applicable, for a period of twelve months after being recruited into the study. Cost analysis was done to quantify cost per procedure, cost of periodontal treatment for the first year and cost per outpatient visit for periodontitis. Analysis was also made according to the different disease severity and treatment status of patients – whether patients received nonsurgical periodontal therapy alone or with surgical therapy, and whether non-surgical rehabilitative dental treatments were also performed for the patients.

We combined two costing methods in this study: the step-down and activity-based costing (ABC) methods, which were substantiated by a clinical pathway. Some of the cost items were costed using the step-down method while others using the ABC. All were added up to provide the total cost. When using the step-down method of costing in a hospital or polyclinic scenario, costs are calculated based on the whole hospital expenditure [21]. This total cost is then allocated to all departments and units using a step-down manner which is based on a definite allocation factor, such as floor space of a particular department or unit or the total number of patients attending the clinics. In this study, the cost of running the specialist periodontal programme is fully allocated to the specialist periodontal clinics as there are no other dental units in the set-up - this provides the final output in this study. The total expenditures are divided by a measure of total output of patient attendances for periodontitis out of patients attending clinics for other periodontal-related conditions, to give “average” cost per patient per outpatient visit. Capital cost (building, equipment ≥ MYR500/€125 per unit,) and some recurrent costs (utilities, maintenance and travelling expenses) were estimated using this approach. Activity-based costing (ABC) is a method of allocating costs to products and services, by assigning costs to all the activities that are used to create them. Items costed using ABC were direct labour costs, equipments ≥ MYR500/€125 per unit and consumables used for each procedure. The eight-member expert group mapped out the usual/standard practices of the whole spectrum of care provided for patients with periodontal disease, and developed a clinical pathway. It was used as a reference to impute total cost of managing periodontal disease from the perspective of health care providers.

### Data collection instruments

We designed three data collection instruments: a costing template for collecting and apportioning administrative and financial data for step-down cost analysis purpose), a form for recording actual equipment, consumables, staff and time taken for each procedure (for ABC cost analysis purpose) and the patients’ diary to measure out-of-pocket expenditures.

### Data analysis

Data were tabulated and calculations made using Microsoft Excel 2010 (Microsoft, Redmont WA USA).

## Results

### Total cost per year of specialist periodontal therapy

In all, 2900 procedures were received by the 165 patients throughout the one-year duration (Table 1). Majority of treatment was the non-surgical periodontal treatment (76.4%), followed by diagnostic procedures (13.6%). About 9% were rehabilitative procedures such as restorations, endodontics and prosthodontics. The least number of procedures was for periodontal surgeries (0.1%). For each patient, the cost of each procedure received was imputed to add up the total cost for the whole study duration. Patient cost was added into the equation for every outpatient visit. The total cost of managing 165 periodontitis patients in one year added up to MYR 465,261(€116, 315) (Table 2). Out of this, 90% was contributed by provider cost and only 10% by patient cost.

**Table 1:** Dental procedures received, by treatment classification

**Treatment classification**	**Number**	**Percentage (%)**
Diagnostics	394	13.6
Non-surgical periodontal treatment	2,215	76.4
Periodontal surgery	26	0.1
Rehabilitative (non-surgical)	265	9.1
All procedures	2,900	100

**Table 2:** Total cost for one year periodontal therapy

	**Provider cost (A)**	**Patient cost (B)**	**Societal cost (A + B)**
Total cost (MYR)	416,431 (€104,108)	48,829 (€12,207)	465,260 (€116,315)
Percentage	90%	10%	100%

### Average cost of periodontitis management at specialist periodontal clinics

The average cost of managing periodontitis patient was MYR 2,820 (€705) per patient per year, and MYR 376 (€94) per outpatient visit (Table 3). As was previously observed, provider cost accounted for nine-fold of patient cost (MYR 2,524/€631) versus MYR 296/€74 per patient per year). Treatment of aggressive periodontitis was significantly higher than of chronic periodontitis (t-test, P = 0.003; Table 4). Moreover, for chronic periodontitis cases, higher cost was expended as disease severity increased (ANOVA, P = 0.022; Table 5). With regard to mix of treatment received, cost was highest for patients who received surgical interventions in addition to non-surgical therapy and lowest for patients who received non-surgical therapy alone (ANOVA, P < 0.001; Bonferroni post-hoc test, P < 0.001; Table 6). Just 19 of the 165 patients, (11.5%) received some form of periodontal surgery. Incidentally, although surgical group IV cost slightly higher than surgical group III, these differences were not statistically significant (Bonferroni post-hoc test, P = 1.000).

**Table 3:** Average cost of periodontitis management

	**Average cost (MYR)**		
	**Provider cost**	**Patient cost**	**Total cost**
Per patient/year	2,524 (€631)	296 (€74)	2,820 (€705)
Per outpatient visit	337 (€84)	40 (€10)	376 (€94)

**Table 4:** Cost of periodontitis management, by type of periodontitis

**Type of periodontitis**	**Number of patients**	**Cost (MYR)**			
		**Mean**	**S.D**	**Median**	**IQR**
Chronic	131	2,636 (€659)	1,459 (€365)	2,375 (€594)	2,191 (€548)
Aggressive	34	3,527 (€882)	1,700 (€425)	3,453 (€863)	2,330 (€583)

Level of significance α = 0.05, t-test, P = 0.003.

**Table 5:** Cost of periodontitis management, by severity of chronic periodontitis

**Disease severity**	**Number**	**Cost (MYR)**			
		**Mean**	**S.D**	**Median**	**IQR**
Mild	8	1,757 (€439)	978 (€245)	1,595 (€399)	1,403 (€351)
Moderate	94	2,545 (€636)	1,499 (€375)	2,234 (€559)	2,170 (€543)
Severe	29	3,174 (€794)	1,277 (€319)	3,103 (€776)	1,958 (€490)

Level of significance α = 0.05, ANOVA, P = 0.022 (Post-hoc Bonferroni test, P = 0.043 for differences between mild and severe periodontitis).

**Table 6:** Cost of periodontitis management, by mix of treatment received

**Mix of treatment**	**Number**	**Cost (MYR)**			
		**Mean**	**S.D**	**Median**	**IQR**
I	72	1,962 (€491)	1,142 (€286)	2,062 (€516)	1,541 (€385)
II	74	3,102 (€776)	1,321 (€330)	2,963 (€741)	2,234 (€559)
III	10	4,847 (€1,212)	1,323 (€331)	4,452 (€1,113)	2,507 (€627)
IV	9	5,103 (€1,276)	1,154 (€289)	5,353 (€1,338)	1,778 (€445)

I – nonsurgical only (NSPT), II – NSPT, nonsurgical rehabilitative therapy (NSRT), III – NSPT, PS and NSRT, IV – NSPT and periodontal surgery (PS).

Level of significance α = 0.05, ANOVA, P < 0.001 (Post-hoc Bonferroni test, P < 0.001 for all pairwise comparisons except for differences between groups III and IV whereby P = 1.00).

### Distribution of cost components

**Figure 3:**
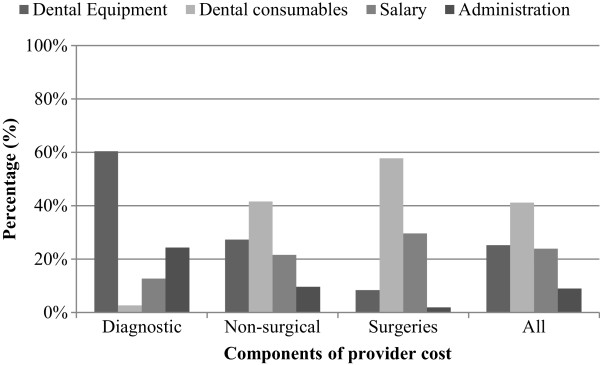
Distribution of provider cost by components.

**Figure 4:**
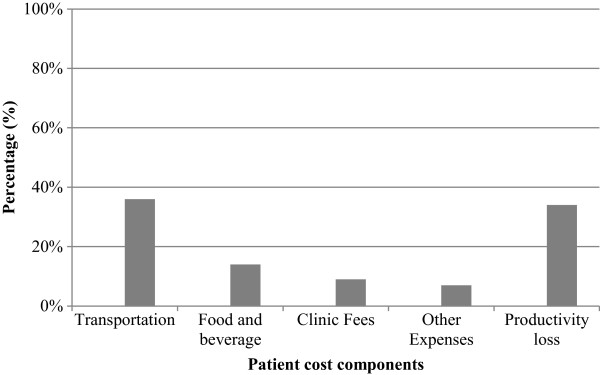
Distribution of patient cost components.

Providers spent an average of 41% of expenditure on consumables, 25% on dental equipment, 24% on salary, and the remaining on administrative work (9%) - Figure 3. A sub-analysis of the cost distribution by type of procedures, however, found that cost of diagnostic procedures may be attributed most to dental equipment (60%) while for the other two procedure categories, consumable items contributed most to total cost (42% for non-surgical and 58% for surgical interventions) - Figure 3. Highest proportion of staff salary was observed for surgical interventions (30%). Patient cost includes money expended on transportation to and from the dental clinic, meals taken, clinic fees (service charges/registration fees) and miscellaneous expenditure related to the dental visit such as payment for crèche, and loss of productivity calculated using the human capital approach. In this study, it was found that patients made 7.5 outpatient clinical visits (range 1 to 17) in the year. Expenditure related to obtaining periodontal treatment was highest on transportation (36%) followed by meals (14%), clinic fees (9%) and other expenses (7%) (Figure 4). Lost productivity accounted for 34% of total patient expenditure.

## Discussion

Despite the significant burden posed by periodontitis on patients and healthcare systems alike, few studies have looked into ways of reducing its impacts, especially in economic terms. Equally critical is the increasing demand for specialist periodontal care, leading to even more rising costs lest the situation is well contained. Hence, optimal allocation of funding and resources becomes necessary to not only ensure best possible outcomes, but to safeguard all segments of care from being unjustly neglected or inappropriately oversubscribed. A cost-of-illness (COI) study provides cost estimates that result from a particular illness or disease. These estimates among others may be used to justify intervention programmes, assist in allocation of resources and provide an economic framework for programme evaluation [19].

In this study, we analysed the cost of managing 165 periodontitis patients up to one-year of active periodontal therapy in specialist periodontal clinics setting. We employed a combination of step-down and activity-based costing methods, and utilized relevant information extracted from a clinical pathway. This approach is deemed to be fitting to cost a range of procedures with various uncertainties in terms of treatment modalities and patient response. One limitation of this study is the relatively short follow-up period which did not capture the whole spectrum of the intended treatment plan, namely surgical interventions. Nonetheless, such circumstances could not be avoided as many factors affect the readiness of the patient to undergo surgery; having acquired good plaque control is one of them and one most challenging to attain.

The average cost of managing periodontitis was estimated to be MYR 2,820 (€705) per patient per year, and MYR 376 (€94) per outpatient visit. Out of the total cost, 96% (MYR 2710/€678) was direct cost. As mentioned earlier this estimation is only for the first year of periodontal therapy, which is the most active phase, whereby the average number of outpatient visits was 7.5 times throughout the year. Following this phase, patients are ideally seen for supportive periodontal therapy or the maintenance phase one to two times a year throughout their lifetime. Modelling of lifelong periodontal treatment cost is beyond the scope of this paper but it may be postulated that cost of subsequent annual treatment could be either (1) lower than the first year, for patients not requiring surgery and provided they remain compliant to home care and follow-up visits, or (2) higher for patients requiring surgery and complex rehabilitative work such as dental implants.

Although patients in this cohort were followed up for only one year, the cost of care incurred was sizeable when compared to the per capita Gross Domestic Product (GDP) of the country and the average annual spending on health per Malaysian. The mean cost of care per patient per admission was 9% of the country’s per capita GDP (MYR 30,856/€7,715) and double of that of the average spending on health per person per year, amounted at MYR 1,296 (€324) as documented in a health care review [25]. This study did not include costing of subsequent phases of periodontal care which comprises the maintenance phase and possible care of medical conditions related to periodontitis. These components of care would increase the total cost of care for these patients and pose a greater burden on the health care expenditure in the country.

The cost for treating aggressive periodontitis was significantly greater than for chronic cases (MYR 3,527/€882 vs. MYR 2,636/€659 respectively). This reflects the more arduous, hence more resource-consuming intervention required for treatment of aggressive periodontitis within the first year. Number of visits required for aggressive cases exceeded those for chronic periodontitis (average of 9.2 times compared to 7.1 times during the study year). For chronic periodontitis cases, average treatment cost was highest for severe periodontitis (MYR 3,174/€794), followed by moderate (MYR 2,545/€636) and mild (MYR 1,757/€439) periodontitis, reflecting a direct relationship between the increased number of outpatient visits with disease severity and the associated resources consumed. This reflection strengthens the dire need for effective primary prevention and/or early detection and treatment of periodontitis; all severe cases started as being mild periodontitis. As such, it would be beneficial to further evaluate existing care pathways and relevant public health promotion strategies to ensure their appropriateness and effectiveness.

Treatment costs were also observed to vary considerably according to mix of treatment patients received. Not unexpected, lowest costs were observed for patients who received only non-surgical treatment while the highest costs were incurred for patients who had received periodontal surgeries. Surgeries include flap curettage, resective as well as regenerative surgeries. Consistently GTR and GBR cost more than just flap surgeries, tunnel preparation, root resections etc. This may be explained by the additional equipment and consumables required on top of the longer treatment time to perform surgical procedures. It is also worth noting that about half of the patients required some form of rehabilitative treatment related to periodontal disease, namely: extractions, restorations, endodontics and prosthodontics. The need to perform some of these procedures at the specialist periodontal clinics was unavoidable, like the need for tooth replacement after extraction of teeth severely affected by periodontitis. High cost of these rehabilitative procedures may be attributed to specialist salary when in fact some procedures, such as removable prostheses, may not require specialists to perform. In addition, teeth requiring restorations could have been treated at primary care level before patients are referred for specialist care. On this note, tooth replacement procedures such as placement of dental implants were not captured during this one-year study but may be the preferred treatment for some patients in the following year.

The absence of COI studies of periodontitis similar with this present one either locally or internationally precludes further comparison. However findings may be discussed in the light of the COI of other chronic conditions. According to Albert and co-workers [26], patients with diabetes mellitus, coronary artery disease and cardiovascular disease who enrolled in an insurance plan who received periodontitis treatment incurred significantly higher medical costs than enrolees who received gingivitis treatment, other dental services, or no dental services. In addition, for middle-aged patients, presence of periodontitis has been shown to increase the inpatient medical care costs for diabetes mellitus, digestive disease, and liver disease [5].

In a study of hypertension outpatients service in a polyclinic in Malaysia, direct cost of treating these patients was reported to be MYR 1,612 (€403), MYR 1,742 (€436) and MYR 2,718 (680) for the pre-hypertensive, stage 1 and stage 2 hypertensive groups respectively [27]. This amount may be considered lower or at most, comparable with direct cost of managing periodontitis patients in this study (MYR 2,710/€678). However, the total indirect costs were much higher for hypertension, which is 73-84% of total cost, specifically: MYR 8,079 (€2,020), MYR 6,655 (€1,663) and MYR 7,511 (€1,878) according to the respective severity of hypertension. In contrast, indirect costs for periodontitis accounted for a meagre 4% of the total cost, reflecting the vast difference both diseases have on loss of productivity. Medications costs were the primary cost driver of the total cost for hypertension while for periodontitis it was the consumables in the form of dental materials. Including medical costs for hypertensive care of periodontitis with hypertension as total costs of managing periodontitis would give a bigger picture of the cost estimates. In this present study, about 28.2% of chronic periodontitis patients had a diagnosis of hypertension – a proportion not to be disregarded.

Comparisons may also be made with medical conditions requiring hospitalised care such as patients admitted for stroke care. In a recent study, the mean cost of care per patient per admission was MYR 3,696 (€924) [28]. Human resources made up the highest cost component (MYR 1,344/€336 or 36% of the total cost), followed by medications (MYR 867/€217) and laboratory services (MYR 338/€85). Length of stay and cost of care varied significantly across different stroke severity levels (p < 0.01). The higher cost of care for stroke compared to periodontitis may be explained by the hospital stay. Elsewhere, a recent review of costs of diabetes care in Malaysia found differences between diabetes patients treated as outpatients at health clinics and as inpatients at hospitals [29]. These findings emphasises that higher costs on inpatient care was mainly due to hospital stay. Nabila and co-workers found direct cost per diabetic patient per year at an outpatient clinic was MYR 186 (€47) for provider cost and MYR 55 (€14) borne by patients [30]. Rohana [31] assessed the cost of diabetes outpatient care for clinics with and without specialist and found that the provider cost per diabetic patient per year for the former was higher (MYR 1,127/€282) than the latter (MYR 802/€201). Meanwhile for inpatient care, Amrizal [32] who measured the provider cost of type 2 diabetic care admitted to medical wards found that MYR 2,161 (€540) was spent per patient per admission. Again, this is comparable to that of periodontitis patients. Furthermore, in the present study, the prevalence of chronic periodontitis diagnosed with diabetes mellitus was 27.8%. Management of these co-morbidities together with the periodontal treatment can be resource intensive, both in terms of routine investigations and medications for the systemic conditions.

Cost estimates from secondary sources which were used in this study were for the following procedures: extraction, dentures, restorations, root canal treatment, crown and bridgework. All these cost estimates were approximated using Ministry of Health’s estimated percentage of government subsidy rates [23] except for crown and bridgework which were based on the recommended scale of private sector fees as determined by the Malaysian Dental Association [24]. It should be noted that MOH’s estimation was done in primary dental care set-ups whereby the dentists’ salary was lower than that of specialists’. This suggests that actual estimate of the cost-of-illness of periodontitis is likely to be higher than the one estimated in this study especially for cases requiring dental rehabilitative treatment.

Patient expenditures account for only one-tenth of the total cost of managing periodontitis. This may be attributed to the low clinic fees paid by these patients – only 75 out of 165 (45.5%) were paying patients, while others qualified for exemption from payment. As such, it is not surprising to find that clinic fees accounted for only 9% of the total patient expenditures. However, patient cost reported in this study (MYR 296/€74) was much higher than the mean out-of-pocket expenses for oral health (MYR 58/€15) for those who sought dental treatment as reported in the National Health and Morbidity Survey [33]. The highest expenditure was on transportation (36%) followed by lost productivity (34%). For most of the states in Malaysia, the public sector specialist periodontal clinic is situated in the city and is responsible for referrals from clinics throughout the whole state. Patients thus may come from distant places and this explains the high proportion of expenses attributed to paying for transportation. Time patients spent on traveling, waiting for treatment and receiving treatment was not measured in this study; instead productivity loss was estimated by calculating their hourly wage for an estimated eight hours’ loss of productivity for each outpatient dental visit that the patient attends. This was assumed appropriate as an average estimation of time spent to receive periodontal treatment at the public sector dental clinic. Indirect cost as measured by patients’ loss of productivity while seeking dental treatment was low, amounting to only 4% of the total cost, including provider cost. In the periodontal literature, patient costs reported usually include expenses paid for the dental treatment received as well as traveling time and no additional component [15,17,34]. Expenses spent for traveling to and from the dental clinic is infrequently measured [35].

One important point to note is that this study also did not include expenses paid for purchasing oral care products. These oral hygiene aids such as toothbrushes, and other mechanical adjuncts such as the dental floss, interdental proximal brush, wood stick and dentifirices have a place in maintaining periodontal health be it in prevention of disease progress or in the treatment of existing disease [36]. Tooth brushing using a manual toothbrush is effective to the extent that it results in reduction of the plaque scores by approximately half. By using an oscillating ⁄rotating toothbrush, additional efficacy can be obtained but the cost could be burdensome for patients who cannot afford it. For periodontal patients who have larger interdental spaces, there is a need to use additional aids for interdental cleaning. Interdental brushes should be the first choice in patients with such open interdental spaces [36]. Meta-analysis showed superiority of the interdental brush to floss with respect to plaque removal. A dentifrice is usually used in combination with tooth brushing; abrasive ingredients have been added to it to enhance the mechanical action of the toothbrush. Purchase of oral hygiene aids may cause considerable financial burden to patients and not all would be likely to use the recommended products no matter how effective, such as the interdental brush.

## Conclusions

We used cost-of-illness approach to estimate cost of managing periodontitis during active periodontal therapy and found the cost to be substantial and comparable with some non-communicable diseases. Total cost comprised 90% of providers’ and 10% patients’ cost. Cost differed by classification of procedures, mix of services received by patient and severity of the periodontal condition. Of all cost components, consumable items generally contributed the most to provider cost, while transportation contributed the most to patient cost. These findings provide basis for identifying potential cost-reducing strategies, estimating economic burden of periodontitis management and performing economic evaluation of the specialist periodontal programme. Some cost-reducing strategies recommended in this paper are to enhance efforts in primary prevention and early detection/treatment of periodontal disease through effective care pathways so that progression of disease may be stopped. Another strategy to reduce cost is to engage general dentists to provide non-specialist dental treatment like supragingival debridement and extraction.

## Competing interests

The authors declare that they have no competing interests in this study.

## Authors' contributions

TMD: principal investigator, project design, data analysis, paper writing and revision. RA: project design, data collection and revision. AM: project design, data analysis, paper writing and revision. MAM: project design, data analysis, paper writing and revision. NI: project design, data collection and revision. YA: project design, data collection and revision. NK: project design, data collection and revision. HA: project design, data collection and revision. ASM: project design, data collection and revision. KA: project design, data collection and revision. SMA: project design, data analysis, paper writing and revision. All authors read and approved the final manuscript.

## Pre-publication history

The pre-publication history for this paper can be accessed here:

http://www.biomedcentral.com/1472-6831/14/56/prepub
